# Protocol for a multicenter prospective sequential intervention study: the effect of adaptive closed-loop postural training on gait disorders in hemiparetic stroke patients

**DOI:** 10.3389/fneur.2026.1752986

**Published:** 2026-04-10

**Authors:** Liping Zhou, Jinping Li, Ying Xu, Xianglian Kang, Jiahui Li, Bo Liang, Bo Chen, Qingjia Guo, Xinzhi Zhang, Zhengfei Wang, Tinghui Huang, Mindan Xu, Zhicheng Bao, Nur Arzuar Abdul Rahim, Ying Hou, Hazwani Ahmad Yusof

**Affiliations:** 1 Department of Community Health, Pusat Kanser Tun Abdullah Ahmad Badawi, Universiti Sains Malaysia, Kepala Batas, Pulau Pinang, Malaysia; 2 Department of Rehabilitation Medicine, The Fourth People's Hospital of Kunshan, Kunshan, Jiangsu, China; 3 Department of Medical Engineering, The Affiliated Suzhou Hospital of Nanjing Medical University, Suzhou Municipal Hospital, Gusu School of Nanjing Medical University, Suzhou, Jiangsu, China; 4 Department of Neurorehabilitation, The Affiliated Suzhou Hospital of Nanjing Medical University, Suzhou Municipal Hospital, Gusu School of Nanjing Medical University, Suzhou, Jiangsu, China; 5 Department of Rehabilitation Medicine, The Affiliated Changshu Hospital of Soochow University, The First People's Hospital of Changshu, Changshu, Jiangsu, China; 6 Department of Rehabilitation Medicine, Gangcheng Rehabilitation Hospital of Zhangjiagang, Zhangjiagang, Jiangsu, China; 7 Department of Rehabilitation Medicine, The Affiliated Kunshan Hospital of Jiangsu University, The First People's Hospital of Kunshan, Kunshan, Jiangsu, China; 8 Department of Clinical Medicine, Pusat Kanser Tun Abdullah Ahmad Badawi, Universiti Sains Malaysia, Kepala Batas, Pulau Pinang, Malaysia

**Keywords:** gait disorders, Gusu Constraint Standing Training, hemiparetic, posture adjustment, stroke rehabilitation

## Abstract

**Introduction:**

Stroke often leads to hemiparetic gait, which negatively affects the quality of life and increases socioeconomic burdens. Conventional therapies have limited effectiveness in improving gait. Postural regulation deficits are a key factor contributing to gait disorders. The Gusu Constraint Standing Training (GCST), a novel posture adjustment approach, shows promise in improving central gait disorders. We hypothesize that GCST optimizes gait in hemiparetic stroke patients by enhancing their postural adaptation criticality and improving gait control through the common neural pathways and nodes shared by posture and gait. However, the specific effects and underlying mechanisms of GCST require further study.

**Methods and analysis:**

This multicenter, prospective, sequential study will recruit 180 hemiparetic stroke patients (recovery phase: 3–6 months; chronic phase: ≥6 months post-stroke) across four hospitals, along with 30 healthy controls. GCST is divided into a structured, stepwise intervention (steps A–E) implemented sequentially based on predefined performance criteria. Assessments will be conducted at baseline, after the completion of each intervention step, and at follow-ups. The primary outcome is the minimal knee flexion angle of the hemiparetic limb during the swing phase, quantified using the three-dimensional gait analysis. Secondary outcomes include gait, postural, neuromuscular, and functional measures. Outcome assessors will be blinded to participants’ stroke phase. Longitudinal data will be analyzed using linear mixed-effects models. Healthy controls will provide normative reference values.

**Ethics and dissemination:**

Approved by each hospital’s ethics committees, the study will require informed consent from participants. The results will be shared through academic conferences and peer-reviewed journals while ensuring participant confidentiality.

**Clincial trial registration:**

https://www.chictr.org.cn/showproj.html?proj=235182, Identifier ChiCTR2400094903.

## Introduction

### Background

Stroke presents a significant challenge in global public health, with its incidence rate increasing (1). Approximately 80% of stroke survivors suffer from gait disorders (2), which further increases the socioeconomic burden. Gait disorders are a common sequela of stroke, often leading to secondary injuries such as falls. Restoring normal gait has become a key rehabilitation goal for stroke patients (3).

Regaining normal gait requires restoring the rhythmic control of the Central Pattern Generator (CPG) for gait (4). A number of therapeutic techniques have been designed and applied to improve motor control on the hemiparetic side, including proprioceptive training, orthotic treatment, and functional electrical stimulation (5, 6). However, these techniques are often ineffective in improving gait rhythm, particularly in terms of dynamic knee control in hemiparetic stroke patients (7). Research on targeted and effective treatments is therefore imperative.

Our team’s previous research has shown that adaptive postural training based on ideal postural alignment can enhance dynamic knee control and gait rhythm (8). Given that posture adjustment and walking share common neural pathways and nodes (9), posture adjustment ability may be a promising approach to enhancing gait patterns. Consequently, we developed the Gusu Constraint Standing Training (GCST), which facilitates automatic gait improvement in patients with cerebral palsy and hemiparesis through targeted postural adjustment training (8, 10). By applying quantified perturbation forces, the GCST induces patients to achieve a critical standing point for maintaining standard postural alignment. This process triggers a transition from quantitative changes to qualitative changes, activates lower-extremity motor integration, and enhances patients’ ability to resist external forces from multiple directions (10, 11).

The GCST improves three-dimensional resistance capacity sequentially in the order of the sagittal, coronal, and horizontal planes. The steps were generally found to improve knee control, optimize the gait cycle, and enhance balance and speed. However, the clinical effects, the mechanisms underlying each step, and how their effects vary according to disease duration remain unclear.

### Significance

The GCST shows promise for improving gait in hemiparetic stroke patients in both the recovery phase and the chronic phase. As the first multicenter sequential intervention study on GCST for these patients, this research will systematically evaluate the GCST’s clinical effects and its mechanisms by assessing five levels, including motor integration, spinal reflexes, muscle control, posture and gait, and quality of life. By comparing hemiparetic stroke patients with healthy adults, the study will analyze gait recovery patterns in these patients. The findings will provide new insights and approaches for restoring lower-extremity neuromuscular function in hemiparetic stroke patients, filling significant gaps in this field.

### Objectives

In hemiparetic stroke patients, postural control deficits are associated with central nervous system lesion sites and disease duration. The GCST enhances standing postural adjustment through sequential, plane-specific resistive adaptive modulation, thereby improving postural adaptation criticality (the dynamic capacity of the neuromuscular system to reorganize postural control under external perturbations). This process also modulates brain motor integration and restores the lower-extremity gait rhythm controlled by the CPG.

The objectives are as follows:

1. To analyze the relationship between postural control deficits and neural damage locations, as well as disease duration, to clarify the specific defects and causes of posture adjustment in hemiparetic stroke patients.
2. To analyze the maximum angles and pulling forces that hemiparetic stroke patients can tolerate in each step and to reveal the adaptive critical state characteristics of postural control.
3. To observe the effects of each GCST step in hemiparetic stroke patients on posture and gait parameters and to systematically assess the clinical efficacy of GCST.
4. To monitor the changes in motor integration in hemiparetic stroke patients by synchronizing multiple muscles of the lower extremities to identify the mechanisms by which GCST optimizes gait patterns through posture adjustment training.

## Methods and analysis

### Study design

**Figure 1:**
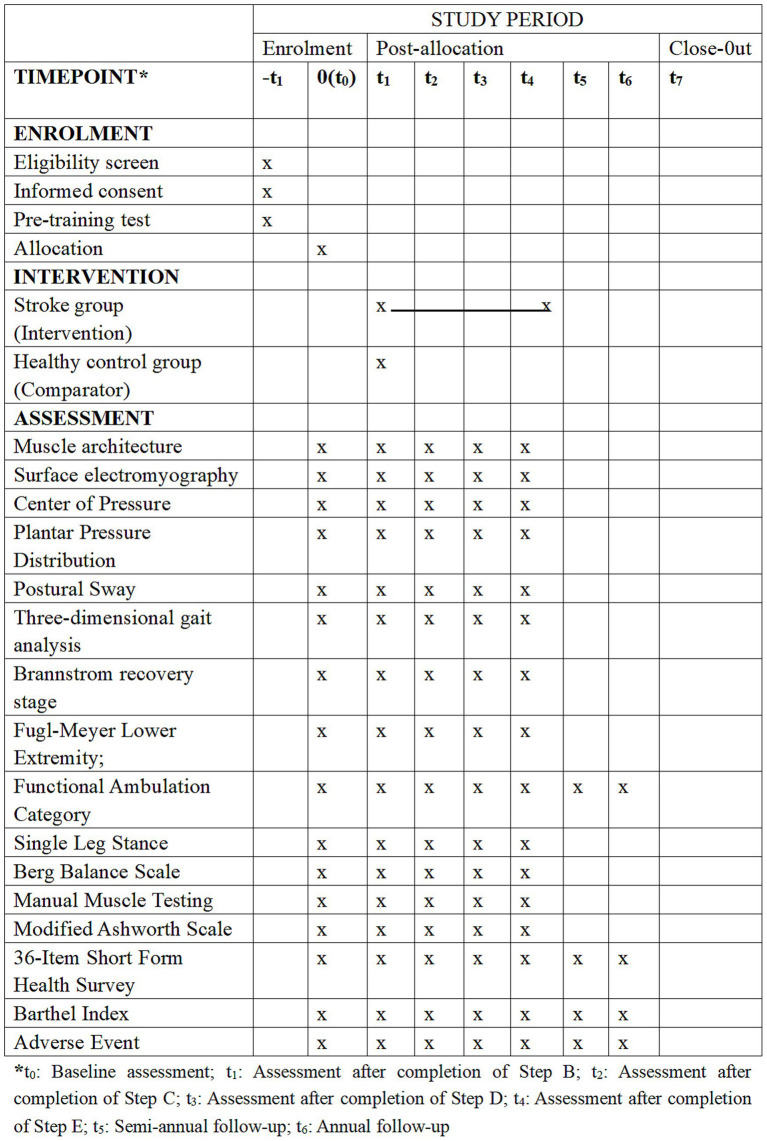
SPIRIT schedule.

**Figure 2:**
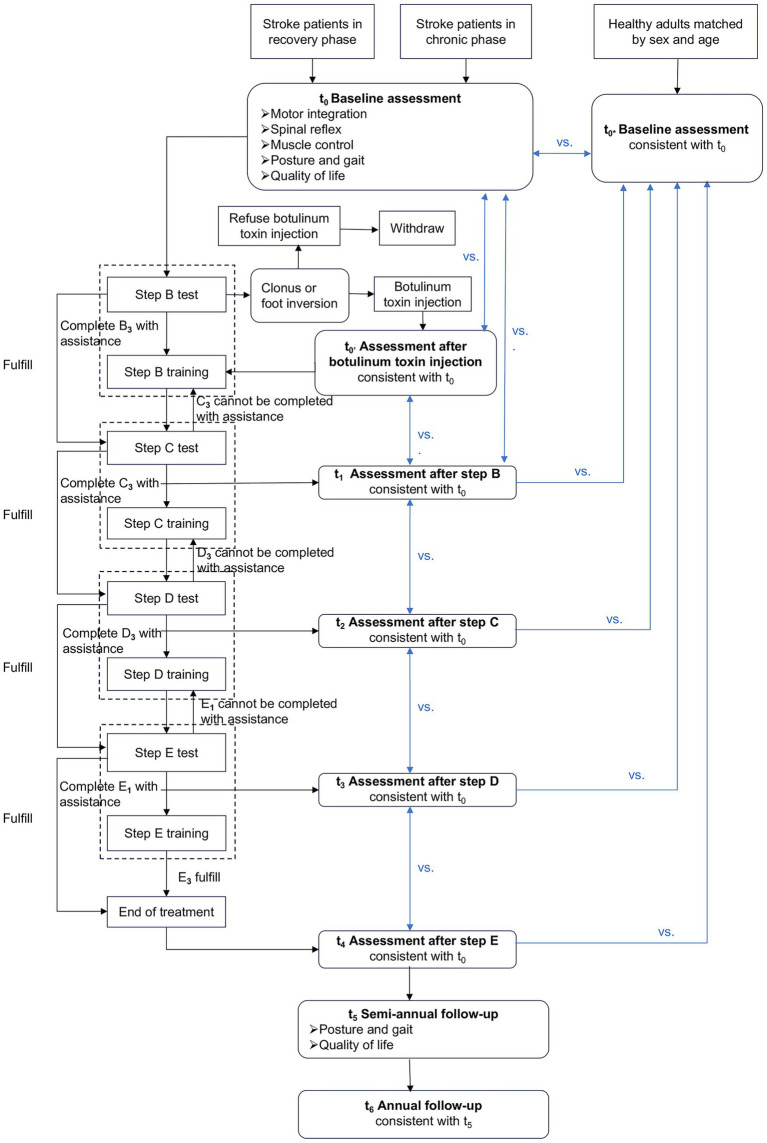
Research flowchart.

This multicenter, prospective, interventional sequential study is led by the Affiliated Suzhou Hospital of Nanjing Medical University. Physicians will recruit hemiparetic stroke inpatients in the recovery phase and the chronic phase from four hospitals in Suzhou—The Affiliated Suzhou Hospital of Nanjing Medical University, The Affiliated Kunshan Hospital of Jiangsu University, The Affiliated Changshu Hospital of Soochow University, and The Gangcheng Rehabilitation Hospital of Zhangjiagang—and 30 healthy adults through the hospitals’ official media. Recruitment is scheduled to begin in October 2025 and will be completed by March 2026. Each participant will be followed for 12 months, so data collection, including follow-up, will conclude in March 2027. Data cleaning and statistical analysis will be conducted from April to June 2027, with primary results expected by July 2027. The study adheres to the SPIRIT guidelines to guarantee high-quality research (12), with the SPIRIT schedule presented in Figure 1 and the specific process illustrated in Figure 2.

The study designates patients in the recovery phase and the chronic phase as the case group and healthy adults as the control group. Comprehensive assessments of patients will be conducted both before and after each GCST treatment step, using data from the healthy adult group as a reference. Time since stroke onset is defined as the interval between the index stroke date and baseline assessment. Participants are categorized into a recovery phase (3–6 months post-stroke) and a chronic phase (≥6 months post-stroke) (13). These time windows correspond to the late subacute and chronic stages of stroke recovery and reflect time-dependent neuroplastic and functional recovery processes (14). A parallel stroke control group receiving standard rehabilitation was not included in the present protocol. This decision was based on the absence of a standardized, consensus-based conventional rehabilitation pathway across participating centers and the highly individualized nature of current clinical practice. Instead, healthy participants were recruited to provide normative reference values for gait biomechanics and neuromechanical indicators. These reference data will be used to characterize recovery trajectories and deviations from physiological patterns in the stroke cohort. In addition, the longitudinal within-subject design and mixed-effects modeling approach will allow each participant to serve as his or her own control over time, partially mitigating the absence of an external stroke comparator.

Given that GCST interventions are implemented in a predetermined sequence, the study does not employ blinding. However, the start and end points of each intervention step are clearly defined by specific tests. The evaluation of treatment efficacy is based on quantitative indicators, which are free from subjective biases. The study adopts a sequential intervention design, allowing patients to commence treatment at a step that matches their functional capabilities and to progress through treatment in a stepwise manner. This approach is advantageous for maximizing the personalized effects of the treatment. To minimize heterogeneity, step skipping is not permitted. All participants are required to complete each step sequentially. Progression is based on predefined functional criteria, and the duration of each step is individually adjusted according to performance. The number of sessions and days required to complete each step are systematically recorded and treated as indicators of intervention exposure.

### Inclusion and exclusion criteria

The inclusion criteria for patient participants in the study are as follows:

1. Patients diagnosed with unilateral cerebral infarction or hemorrhage by CT or MRI;
2. First occurrence of stroke with a clearly documented stroke onset date and currently in either the recovery phase (3–6 months post-stroke) or the chronic phase (≥6 months post-stroke) at the time of enrollment (15);
3. Patients aged between 40 and 70 years;
4. Those able to cooperate during examinations and follow simple instructions;
5. Those exhibiting lower-extremity motor function impairment and hemiparetic gait;
6. Those with the ability to stand independently for 2 min (provide stable center-of-pressure measurements in static balance assessments) (16) or to complete more than five consecutive gait cycles to ensure reliable kinematic data in three-dimensional gait analysis (17); and
7. The non-affected side has no significant coordination impairments and has muscle strength above grade 4 (ensuring the ability to participate in weight-bearing postural training while minimizing fall risk) (18).

The exclusion criteria for patient participants in the study are as follows:

1. Cerebellar injury or significantly reduced muscle tone.
2. History of lower-extremity nerve, muscle, or skeletal system diseases or surgeries.
3. Presence of lower-extremity joint pain or exhibiting significant foot inversion, clonus, or requiring botulinum toxin injection prior to training.
4. Currently participating in other clinical trials.
5. Severe diseases such as high-risk epilepsy or major cardiac conditions.
6. Poor compliance or inability to complete the study interventions.

The healthy control group will be matched to the stroke patients for sex, age, and body type. They must be free of lower-extremity nerve, muscle, or skeletal diseases; pain; or surgery, which ensures their data reflects a healthy state.

### Sample size estimation

The sample size calculation was based on an internal pilot dataset (n = 30) collected at the coordinating center prior to trial registration, using the minimal knee flexion angle during the swing phase on the hemiparetic side as the primary outcome measure. The pilot study was approved by the Medical Ethics Committee of the Affiliated Changshu Hospital of Soochow University (Approval No. X202265), and pilot participants were not included in the current trial (19). The use of pilot data to inform feasibility and parameter estimates for subsequent studies is supported by methodological guidance on pilot studies and pilot-informed trial planning (19, 20).

In the pilot dataset, the mean pre–post change in the primary outcome was 1.2 degrees, with a standard deviation of the within-subject change score of 6.36. The standardized effect size was therefore d = 0.1887 (d = Δμ/SDΔ). Although the pilot-derived effect size was small, the minimal knee flexion angle represents a joint-level biomechanical marker of neuromuscular coordination (21). Variations in swing-phase knee flexion primarily reflect changes in limb advancement mechanics and inter-joint coordination rather than global functional performance. Therefore, even relatively small angular differences may provide meaningful biomechanical information relevant to post-stroke gait recovery. Sample size estimation was performed using G*Power (difference between two dependent means, matched pairs). With an effect size (dz) of 0.1887, a one-sided significance level (*α*) of 0.05, and statistical power (1−β) of 0.80, the required minimum sample size was calculated to be 174 patients. A one-sided test was adopted, given the directional hypothesis that GCST would improve, rather than worsen gait performance. To account for potential attrition and to ensure balanced recruitment across centers, the planned recruitment target was set at 180 participants across four participating centers (approximately 45 participants per center). Based on pilot observations and adherence-enhancing procedures implemented in the study protocol, the anticipated attrition rate is expected to remain low. Healthy controls were not included in the sample size calculation and were recruited solely to provide normative reference values.

### Patient and public involvement

The patients and public involvement informed the study’s development, integrating feedback to align the design, methods, and implementation with the needs of the patients and the public. During pilot implementation, informal feedback was obtained from stroke patients regarding session tolerance and assessment burden. Participants reported increased fatigue during prolonged standing tasks and expressed a preference for structured rest intervals. In response, the session duration was standardized within a tolerable range. The order of biomechanical assessments was also adjusted to minimize cumulative fatigue.

### Intervention

Physicians will enroll hemiparetic stroke patients in the recovery and chronic phases for GCST treatment. Healthy controls will undergo a single assessment session without intervention. Healthy controls are included to establish normative reference values for gait kinematics and neuromuscular parameters. Their data will serve as a benchmark for quantifying baseline deviations from typical gait patterns in the stroke cohort and for contextualizing longitudinal changes observed during follow-up. Changes in the stroke cohort will be compared with normative values to evaluate the degree to which gait parameters converge toward typical movement patterns over time.

**Figure 3:**
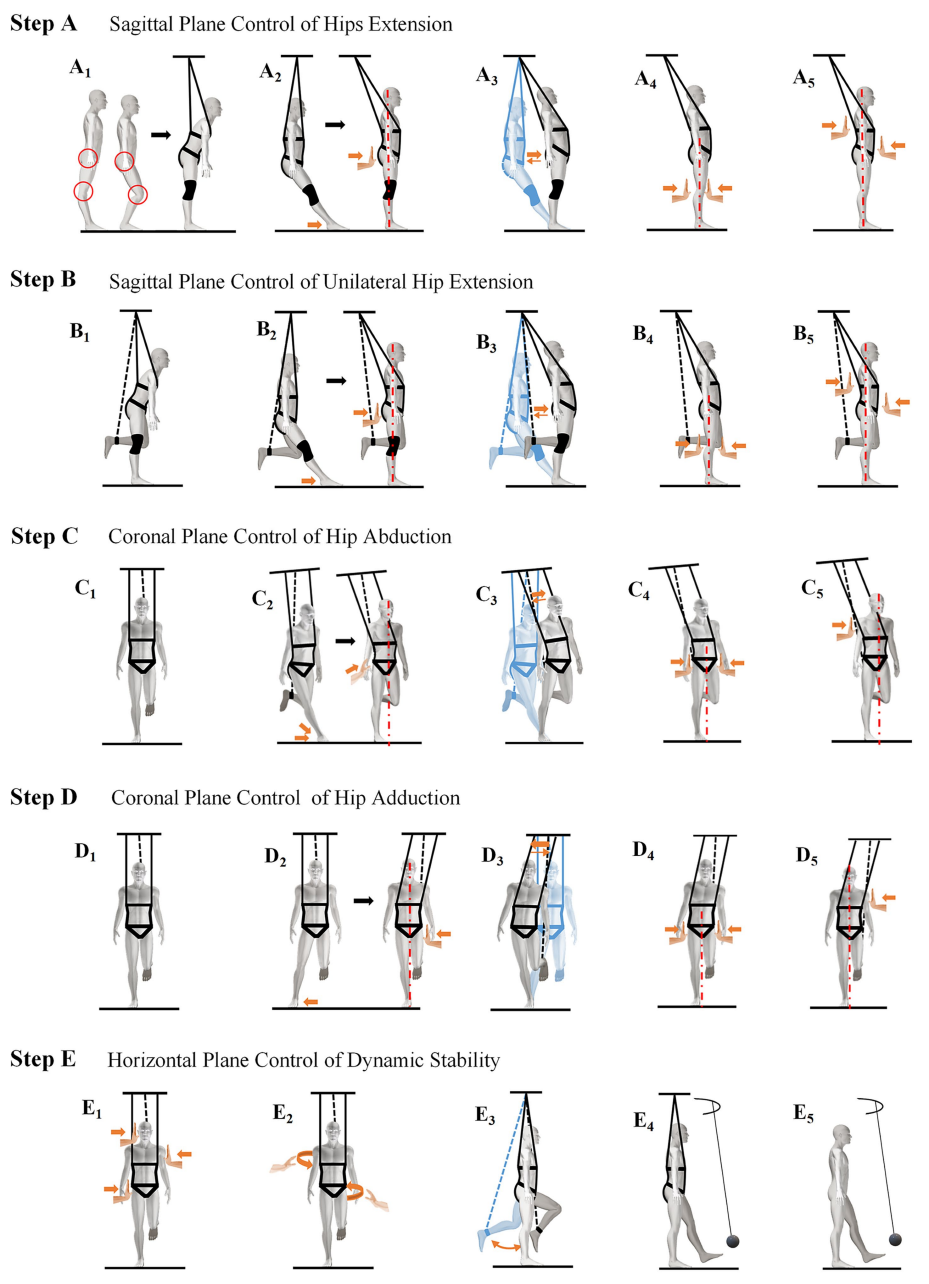
Diagram of the GCST steps.

All participating therapists must have at least 3 years of clinical experience in neurological rehabilitation. Prior to study initiation, therapists will undergo a centralized GCST training workshop including theoretical instruction, demonstration of each intervention step (A–E), supervised practice sessions, and competency assessment. Certification requires the successful completion of a standardized checklist and the demonstration of correct procedural execution. Refresher training sessions are conducted quarterly. Therapists will administer the GCST program, which involves 30-min daily sessions, five times per week. The GCST steps are illustrated in Figure 3.

Given that all participants are unilateral hemiparetic, the training commences at step B. Each step utilizes overlapping motor tasks and incremental difficulty to progressively unlock the patients’ potential. Progression from one GCST step to the next is based on predefined performance criteria rather than therapist discretion. Upon passing the step E test, the treatment cycle is concluded. Throughout the treatment, the number of days taken to complete each step is recorded. Each step is implemented at the patient’s maximum tolerable intensity. Once the criteria for progression are met, the next step is initiated immediately. Specific operational details are provided in Figure 2. Intervention sessions are terminated if participants experience dizziness, excessive fatigue (Borg scale > 15), unstable blood pressure, pain exacerbation, or an inability to maintain a safe standing posture. Safety monitoring is performed continuously during training. To ensure treatment fidelity across centers, a standardized intervention manual has been developed. Sessions are documented using structured fidelity checklists. A random 10% of sessions are video recorded (with patient consent) and reviewed by an independent senior investigator. Deviations from the protocol are documented, and corrective feedback is provided.

All hemiparetic stroke patients will receive conventional treatments based on clinical guidelines. They can participate in upper-extremity training, sitting balance exercises, lying-position training, stretching programs, and physical therapy as needed. However, strength training or motor skill training related to standing and gait functions is expressly excluded.

### Assessment and follow-up

Biomechanical assessment equipment is calibrated according to manufacturer guidelines prior to each testing session. Calibration logs are maintained at each center to ensure measurement consistency. Assessments will be conducted at baseline, after the completion of each step, and during semi-annual and annual follow-ups post-treatment. The primary outcome of this study is the minimal knee flexion angle of the hemiparetic limb during the swing phase of gait, measured by three-dimensional gait analysis. This parameter is defined as the lowest knee flexion value observed in the swing phase of each gait cycle and reflects dynamic knee extension control during limb advancement. Reduced knee flexion during swing is a defining biomechanical feature of post-stroke stiff-knee gait and has been consistently associated with impaired toe clearance, altered limb advancement, and compensatory strategies such as hip circumduction and contralateral vaulting (22, 23). From a neuromechanical perspective, insufficient swing-phase knee flexion is linked to abnormal muscle activation patterns, particularly prolonged quadriceps activity and disrupted inter-joint coordination, making this parameter sensitive to lower-limb neuromuscular integration and rehabilitation-induced changes (21, 24) Table 1 presents a detailed schedule of the assessments and the responsible centers.

**Table 1:** Schedule of assessments and measurement time points.

Timepoint* Assessments^#^	t_0_, t_0*_	t_1_	t_2_	t_3_	t_4_	t_5_	t_6_
MA	√○□	√○□	√○□	√○□	√○□		
sEMG	√	√	√	√	√		
COP	□	□	□	□	□		
PPD	√□	√□	√□	√□	√□		
PS	□	□	□	□	□		
3DGA	√○	√○	√○	√○	√○		
BRS	√○□	√○□	√○□	√○□	√○□		
FMA-LE	√○□	√○□	√○□	√○□	√○□		
FAC	√○□	√○□	√○□	√○□	√○□	√○□	√○□
SLS	√○□	√○□	√○□	√○□	√○□		
BBS	□	□	□	□	□		
MMT	√○□	√○□	√○□	√○□	√○□		
MAS	√○□	√○□	√○□	√○□	√○□		
SF-36	√○□	√○□	√○□	√○□	√○□	√○□	√○□
BI	√○□	√○□	√○□	√○□	√○□	√○□	√○□
TEAE		√○□	√○□	√○□	√○□	√○□	√○□

Time points: t_0_: Baseline (patients); t_0*_: baseline (healthy controls); t_1−_ t_4_:post-step assessments (after the completion of steps B–E, respectively); t_5_: semi-annual follow-up; t_6_: annual follow-up. MA, muscle architecture; sEMG, surface electromyography; COP, center of pressure; PPD, plantar pressure distribution; PS, postural sway; 3DGA, three-dimensional gait analysis; BRS, Brannstrom recovery stage; FMA-LE, Fugl–Meyer Lower Extremity; FAC, functional ambulation category; SLS, single-leg stance; BBS, Berg Balance Scale; MMT, manual muscle testing; MAS, Modified Ashworth Scale; SF-36, 36-Item Short Form Health Survey; BI, Barthel Index; TEAE, treatment-emergent adverse event; Center indicators: √, Suzhou and Kunshan sites; ○, Changshu site; □, Zhangjiagang site.

### Outcome measures

1. Muscle architecture

Muscle thickness and structural characteristics of 22 lower-extremity muscles will be assessed using high-frequency ultrasound (X-Porte TTC). These muscles include bilateral gluteus maximus, gluteus medius, gluteus minimus, rectus femoris, vastus medialis, vastus lateralis, vastus intermedius, popliteus, long head of the biceps femoris, short head of the biceps femoris, semitendinosus, and semimembranosus. Restoration of muscle thickness symmetry has been shown to correlate with improved muscle strength (25).

2. Surface electromyography indicators

Using the Noraxon Ultium wireless surface electromyography system (Noraxon, Scottsdale, AZ, USA), in accordance with European Surface EMG for non-invasive assessment of muscles (SENIAM) guidelines (26), 16 wireless sEMG electrodes are placed on the bilateral gluteus maximus, gluteus medius, rectus femoris, vastus medialis, biceps femoris, tibialis anterior, medial gastrocnemius, and soleus muscles. Signals are synchronously recorded at a sampling rate of 2,000 Hz. A fourth-order Butterworth bandpass filter (20–500 Hz) is applied to eliminate low-frequency noise and high-frequency interference, and a notch filter is used to remove 50 Hz power-frequency interference. Data are collected during the Achilles tendon reflex, standing, and walking tasks to calculate muscle frequency-domain characteristics and non-linear network indicators (11).

3. Postural parameters

Center of Pressure: A balance test and training device (ProKin 254, TecnoBody, Italy) is utilized to assess balance during bipedal stance. The static load test is conducted with a three-dimensional force platform (Advanced Mechanical Technology, Inc., BMS400600, USA) to quantify forces and torques in a stationary state.

Plantar Pressure Distribution: A pressure distribution measurement platform (Sensor Medica, FM30050, Italy) is utilized to measure pressure distribution, contact area, and center position.

Postural Sway: The APDM system (Opal sensors, Mobility Lab v2) is used to measure the postural sway during standing with eyes open and closed. Sensors are fixed on both wrists, the dorsum of both feet, the xiphoid process of the sternum, and the L_5_ spinous process for a 30-s test (27). It can detect 33 postural-sway parameters, including 25 acceleration parameters and 8 angular parameters.

4. Gait parameters

Three-Dimensional Gait Analysis: A three-dimensional motion capture system (Vicon, Vero 2.2, UK) is used to collect kinematic and dynamic parameters during walking. Reflective markers are placed on the body based on the Full-Body Gait Model (28). Data collection is performed using Vicon Nexus. After data collection, the Polygon report analysis software is used to calculate fundamental spatiotemporal parameters, joint angles, and moments during gait, focusing on key joints such as the knees and hips. A three-dimensional force platform (Advanced Mechanical Technology, Inc., BMS400600, USA) captures real-time force and pressure changes throughout the gait cycle, enabling the calculation of joint moments, joint forces, and dynamic characteristics of gait (29). Simplified gait tests using the APDM system (Opal sensors, Mobility Lab v2) are conducted to detect 40 gait parameters, including 4 arm parameters, 6 axial parameters, 20 lower-extremity motion parameters, and 10 gait cycle ratios.

5. Lower-extremity motor function

The Brunnstrom Recovery Stage assessment is used to assess motor recovery in post-stroke patients, dividing the recovery process into stages from no spontaneous movement to the ability to perform complex movements independently (30). The Fugl–Meyer Lower-Extremity scale evaluates lower-extremity motor control in post-stroke patients, covering strength, muscle tone, range of motion, and coordination. An FMA-LE score of 21 or higher indicates a higher level of functional mobility (31), while an increase of 6 points suggests a significant improvement in rehabilitation outcomes (32). The Functional Ambulation Category assesses patients’ walking ability in various environments to determine their independence and stability in daily activities (33). Single-leg stance quantifies balance ability by recording the longest standing time over three trials, performed under both eyes-open and eyes-closed conditions (34). The Berg Balance Scale, which includes 14 dynamic and static tasks, comprehensively assesses balance function. A total score below 40 indicates a risk of falls (35). Manual Muscle Testing is used to evaluate the strength of the gluteus maximus, gluteus medius, quadriceps, hamstrings, tibialis anterior, gastrocnemius, and soleus muscles using a 5-point scoring system (36). The Modified Ashworth Scale assesses muscle tone of the knee extensors and ankle plantar flexors, with 0 indicating no resistance and 4 indicating significant stiffness during flexion or extension (37).

6. Quality of life

The 36-Item Short Form Health Survey is a highly reliable and valid questionnaire that is widely accepted for its ease of use. It includes eight dimensions, such as physical functioning, social functioning, pain, and vitality, making it suitable for evaluating quality of life across various diseases (38). The Barthel Index measures functional independence in activities of daily living. It comprises 10 items related to daily living activities, with a total score of 100 points; higher scores indicate greater independence in daily life (39).

### Adverse events and safety

This study strictly adheres to relevant standards to ensure the compliance of equipment and personnel with these standards. Therapists will provide professional supervision throughout the training to minimize accident risks. Despite extensive preventive measures, potential risks such as falls, ankle sprains, soft tissue edema, and seizures remain. To address these, an emergency response plan has been developed, and risks such as equipment damage have been considered.

All adverse events and unexpected reactions during the study will be promptly recorded and assessed. In the event of an adverse event, participants’ changes in condition will be immediately reported, and necessary treatment will be provided until stability is achieved. The research team will evaluate the relationship between adverse events and the treatment.

### Participant compliance

The following methods are implemented in this study to guarantee the validity of the experimental data and enhance participant compliance:

1. All trial protocols, procedures, and requirements are clearly explained to participants and researchers, along with detailed operational guidelines.
2. Comprehensive training is provided for participants and researchers to ensure a clear understanding of the study’s objectives, roles, and responsibilities.
3. Regular supervision, feedback, and guidance are established to assist therapists in tackling challenges during training.
4. Assessment schedule reminders are sent via email and phone to participants, with flexible arrangements to maintain data collection continuity and integrity.

### Data collection and management

Primary outcome indicators will be collected using standardized instruments to ensure data accuracy and objectivity. Secondary outcome indicators will be assessed independently by two professional therapists, with results cross-verified. Any discrepancies will be discussed to reach a consensus.

For patients who withdraw or change their training regimen, the time and reasons for withdrawal will be documented to determine whether their data will be included in the final analysis. If corrections are needed for original records, a dated statement explaining the changes must be attached. All original study documents and copies will be stored for inspection.

Data entry will use an electronic data capture system with double data entry to minimize errors. Each participant’s electronic case report form will be backed up. Project administrator accounts will be created to ensure that researchers have appropriate data access permissions based on their roles, responsibilities, and confidentiality agreements. Data uploaded from each center will be monitored by a third-party quality control organization (The Fourth People’s Hospital of Kunshan).

### Statistical analysis

Statistical analysis will be performed using SPSS software (V.26.0). Data normality will be assessed using the Shapiro–Wilk test, and variance homogeneity will be evaluated using Levene’s test. Normally distributed continuous variables will be expressed as mean ± standard deviation, and non-normally distributed continuous variables will be presented as median (interquartile range). Descriptive statistics will be used to summarize baseline demographic and clinical characteristics.

All statistical analyses will be conducted using linear mixed-effects models (LMMs) to account for repeated measurements, individualized intervention exposure, and inter-individual variability. Participant identity will be included as a random intercept, and time (baseline, post-intervention, follow-up) will be specified as a fixed effect. The primary outcome will be modeled within this framework. For secondary outcomes and exploratory analyses involving multiple comparisons, the Holm–Bonferroni correction will be applied to control the family-wise error rate. The primary outcome will not be adjusted for multiplicity.

Stroke phase (recovery vs. chronic) and center will be included as fixed effects. Time-by-phase interactions will be examined to explore potential differences in longitudinal recovery trajectories between stroke phases. To address heterogeneity in intervention exposure inherent to the sequential adaptive design, cumulative training dose and step-specific duration will be incorporated as time-varying covariates. This approach allows adjustment for individual differences in progression speed and treatment intensity. In the presence of non-normal outcome distributions or convergence difficulties, generalized estimating equations (GEEs) with appropriate link functions will be conducted as sensitivity analyses to assess the robustness of the primary findings.

Missing data will be handled under the missing-at-random assumption using the maximum likelihood estimation inherent in LMMs, which allows inclusion of participants with incomplete follow-up data without listwise deletion. Sensitivity analyses will be conducted to assess robustness. Effect sizes will be reported as model-estimated mean differences with 95% confidence intervals. Standardized effect sizes (Cohen’s d) will also be calculated where appropriate to facilitate clinical interpretation.

## Ethics and dissemination

This trial is registered at the Chinese Clinical Trial Registry (ChiCTR, https://www.chictr.org.cn) with the reference number ChiCTR2400094903. All participants will provide written informed consent on a form detailing the study’s purpose, methods, procedures, risks, and benefits. The consent form also informs participants of their rights and obligations and provides the principal investigator’s contact number. Participation is voluntary, and withdrawal at any time will not result in penalties or loss of benefits. The study has been approved by the Ethics Committee of Nanjing Medical University Gusu College (approval number K-2024-132-H01), which will handle all ethics-related issues during the study. The study will strictly adhere to relevant regulations to ensure the ethical compliance of equipment and personnel. A system for monitoring and reporting serious adverse events will be established, and data privacy and confidentiality will be ensured. Participants will not receive financial compensation for participation. When applicable, reasonable transportation expenses will be reimbursed to minimize economic burden.

All data will be anonymized using unique study identification codes. Personally identifiable information will be stored separately from research data in a secure database with restricted access. Electronic data will be maintained on password-protected institutional servers, and paper-based documents will be stored in locked cabinets at the coordinating center. Only authorized research personnel will have access to identifiable data. Data handling procedures comply with institutional regulations and applicable data protection standards. The study results will be published in peer-reviewed journals and presented at medical conferences. Additionally, the results will be submitted to public clinical trial databases to ensure transparency and openness.

## Discussion

This multicenter, prospective, sequential study assesses the GCST’s efficacy in optimizing hemiparetic stroke patients’ gait and its underlying mechanisms. Conducted across general hospitals, the study enhances sample heterogeneity and representativeness and boosts the clinical applicability of its findings.

A key objective is to clarify the deficits and causes of posture adjustment in hemiparetic stroke patients. Postural regulation, the ability to dynamically adjust muscle tone to maintain balance during movement, is fundamental for motor and behavioral control. It is governed by a hierarchical neural control system spanning from the cerebral cortex to the spinal cord and is often impaired in patients with central neural damage. This impairment affects gait and extremity coordination, which rely on intact postural regulation. Conventional posture adjustment assessments typically focus on stability rather than directional control. This study innovatively uses the time patients maintain standard alignment under external force interference as a criterion to evaluate critical posture adjustment ability. This method is expected to more accurately reflect the posture adjustment ability of patients in a non-compensatory state. Sequential interventions allow the observation of GCST’s impact on coordination, posture, and gait recovery, helping to determine treatment benefits and prognosis in patients with varying stroke characteristics.

Another objective is to evaluate the GCST’s clinical effectiveness. Previous research indicates that the angle of force application in GCST significantly affects muscle recruitment (10). To achieve comprehensive muscle activation, patients must complete multidimensional GCST steps ranked by difficulty, with intervention progressing step by step from the sagittal plane, through the coronal plane, to the horizontal plane. This approach may activate the brain’s sensory-motor cortex, thereby promoting motor learning (40) and enhancing postural regulation.

The study also aims to explore the mechanisms through which GCST improves gait control via posture adjustment training. Posture and gait share common neural pathways, including the brainstem, the cerebellum, the basal ganglia, the spinal central pattern generator, and pathways such as the reticulospinal and vestibulospinal tracts (4). Both systems rely on integrating sensory feedback and motor commands for efficient movement (9, 41). The GCST targets posture adjustment to activate lumbar spinal CPG functions (4), facilitating a rhythmic gait.

The GCST targets overall lower-extremity control. Conventional muscle assessments often fail to capture the overall postural treatment effects. Our team innovatively performed dynamic network analyses on surface electromyography signals for multiple muscles and developed a surface electromyography index for multi-muscle coordinated control. These indices can accurately detect abnormalities in muscle coordination, including paralysis and compensatory movement strategies, as well as assess the coordination of individual muscles during movement (11), thereby enabling a precise evaluation of lower-extremity motor integration.

In summary, the GCST is supported by a solid theoretical and practical foundation. This study establishes a basis for its clinical application and may provide new insights into lower-extremity functional impairments and recovery mechanisms in stroke patients.
